# Bioactive Compounds, Antioxidant, and Antibacterial Properties of *Lonicera caerulea* Berries: Evaluation of 11 Cultivars

**DOI:** 10.3390/plants10040624

**Published:** 2021-03-25

**Authors:** Laima Česonienė, Juozas Labokas, Ina Jasutienė, Antanas Šarkinas, Vilma Kaškonienė, Paulius Kaškonas, Rita Kazernavičiūtė, Aistė Pažereckaitė, Remigijus Daubaras

**Affiliations:** 1 Botanical Garden, Vytautas Magnus University, LT-46324 Kaunas, Lithuania; remigijus.daubaras@vdu.lt; 2 Laboratory of Economic Botany, Nature Research Centre, LT-08412 Vilnius, Lithuania; juozas.labokas@gmail.com; 3 Food Institute, Kaunas University of Technology, LT-50254 Kaunas, Lithuania; ina.jasutiene@ktu.lt (I.J.); antanas.sarkinas@ktu.lt (A.Š.); 4 Department of Food Science and Technology, Kaunas University of Technology, LT-50254 Kaunas, Lithuania; rita.kazernaviciute@ktu.lt (R.K.); aiste.pazereckaite@ktu.edu (A.P.); 5 Faculty of Natural Sciences, Instrumental Analysis Open Access Centre, Vytautas Magnus University, LT-44404 Kaunas, Lithuania; vilma.kaskoniene@vdu.lt; 6 Institute of Metrology, Kaunas University of Technology, LT-51368 Kaunas, Lithuania; paulius.kaskonas@ktu.lt

**Keywords:** blue honeysuckle, cultivar, berry extract, bioactive compound, phenolics, anthocyanins, saccharides, organic acid, radical scavenging capacity, antibacterial activity

## Abstract

The aim of the study was to evaluate 11 cultivars of blue honeysuckle (*Lonicera caerulea* L.) for bioactive compounds, antioxidant capacity, and the antibacterial activity of berries. Total phenolic contents (TPCs) and total anthocyanin contents (TACs) were established by using ethanolic extracts. For contents of organic acids and saccharides, aqueous extracts were used, and vitamin C was determined by using oxalic acid solution. DPPH^•^ radical scavenging capacity was evaluated by using ethanolic extracts; antibacterial activity was assessed by using both ethanolic and aqueous extracts. The TPC varied from 364.02 ± 0.41 mg/100 g in ‘Vostorg’ to 784.5 ± 0.3 mg/100 g in ‘Obilnaja’, and TAC ranged from 277.8 ± 1.1 mg/100 g in ‘Čelnočnaja’ to 394.1 ± 8.4 mg/100 g in ‘Nimfa’. Anthocyanins comprised 53.8% of total phenolic contents on average. Among organic acids, citric acid was predominant, averaging 769.41 ± 5.34 mg/100 g, with malic and quinic acids amounting to 289.90 ± 2.64 and 45.00 ± 0.37 mg/100 g on average, respectively. Contents of vitamin C were 34.26 ± 0.25 mg/100 g on average. Organic acids were most effective in the inhibition of both Gram-positive and Gram-negative bacteria tested. In conclusion, berries of *L. caerulea* are beneficial not only for fresh consumption, but also as a raw material or ingredients of foods with high health-promoting value.

## 1. Introduction

Along with nutritional values, berries provide key benefits to human health because they contain vitamins, phenolics, volatile organic compounds, organic acids, and other bioactive compounds needed for the normal functioning of our bodies. This is the reason why fresh and processed berries, particularly those rich in certain bioactive compounds, are in high market demand. It was even proposed that fruits which contain powerful bioactive compounds, characterized by high antioxidant capacity such as polyphenols, anthocyanins, or procyanidins, may be classified as superfruits [1], thus emphasizing their role in human health and diet. Irrespective of how they are classified, the search for promising fruit and berry crops has been increasing along with the development of appropriate laboratory techniques and globalization. One such relatively new berry crop is *Lonicera caerulea*, or blue honeysuckle, family Caprifoliaceae Juss.

The tetraploid species *Lonicera caerulea* L. belongs to the Lonicera *L. subsect*. *Caeruleae* Rehd. [2]. The first experiments on the domestication of this species commenced in Russia in 1913–1915. Genotypes for the breeding of new cultivars were collected in northern and eastern Russia, Japan, and China, where the richest genetic diversity was concentrated in natural habitats [2,3]. Comprehensive investigations on the biological properties of *L. caerulea* corroborated the early ripening of berries, winter hardiness, and high resistance to spring frosts [4]. Several studies reported that berry yields of different wild genotypes and cultivars varied from 0.26–1.24 kg/bush [5], from 977–2216 g/bush [6], and from 0.478–1.873 kg/bush [7].

Berries of *L. caerulea* are distinguished by the content of biologically active substances, which depends on climatic conditions and cultivation techniques [8,9]. A high total polyphenolic content was reported as an important property of blue honeysuckle berries [10], while it is known that phenolic compounds are among the most widespread bioactive substances associated with the antioxidant activity of different fruits. In general, the relationship between high contents of phenolic compounds in different berries and their high biological activities are widely acknowledged [11,12,13]. Different studies have confirmed that certain types of phenolic compounds are variety-dependent and show greater antioxidant activity than others [14,15]. It has been established that berries of *L. caerulea* accumulate flavonols (quercetin, quercitrin, rutin) as well as flavanes (catechins, proanthocyanidins) [12,16]. It also has been confirmed that anthocyanins, conditioning the blue color of berries, are very strong antioxidants, important for human health [17]. As Del Bo et al. [18] have reported, anthocyanins constitute large proportions of berry polyphenols. Therefore, berries of *L. caerulea* are appreciated as an excellent source of anthocyanins which are widely used in both the food and pharmaceutical industries [14,19,20]. Chemical analysis of *L. caerulea* berries revealed that cyanidin-3-O-glucoside is the major anthocyanin, comprising 79–92% of the total anthocyanin content and over 60% of the total content of polyphenolic compounds [21].

Several studies have reported the strong antioxidant activities of fresh *L. caerulea* berries, berry juices, and extracts [13,19,22]. Recent studies on antimicrobial properties have also demonstrated the high activity of bioactive compounds found in these berries [21,23,24].

Higher levels of ascorbic acid were reported in *L. caerulea* berries compared to strawberries, blueberries, and raspberries [25,26,27]. These berries are also valued as a source of organic acids and carbohydrates, mostly sugars, the ratios of which define the taste of berries. Different amounts of citric, malic, shikimic, quinic, and tartaric acids were reported in berries of *L. caerulea* [20,28]. Monosaccharides, fructose and glucose, were established to be predominant in honeysuckle berries [9]. It is important to note that berries of approved cultivars of *L. caerulea* are appreciated by consumers for their pleasant sweet and sour taste. This is one of the reasons for this berry plant becoming more popular in farms, plantations, and gardens in Poland, Czech Republic, Slovakia, Austria, Germany, and Canada [29]. However, berries produced from seed-grown plants or propagated from the wild genotypes may have an unacceptably bitter taste. Thus, for the breeding of new cultivars, it is critical to select the right genotypes producing fruit not only with high levels of bioactive compounds but also distinguished by taste qualities.

The aim of this study was to evaluate 11 cultivars of blue honeysuckle (*Lonicera caerulea* L.) for bioactive compounds, antioxidant capacity, and the antibacterial activity of berries. The cultivars were evaluated for the total phenolic contents, total anthocyanins, saccharides, organic acids, vitamin C, pH, and dry matter contents of berries.

## 2. Results and Discussion

### 2.1. Variation in Ripening Time, Berry Size, pH, and Dry Matter (DM) Content

**Figure 1:**
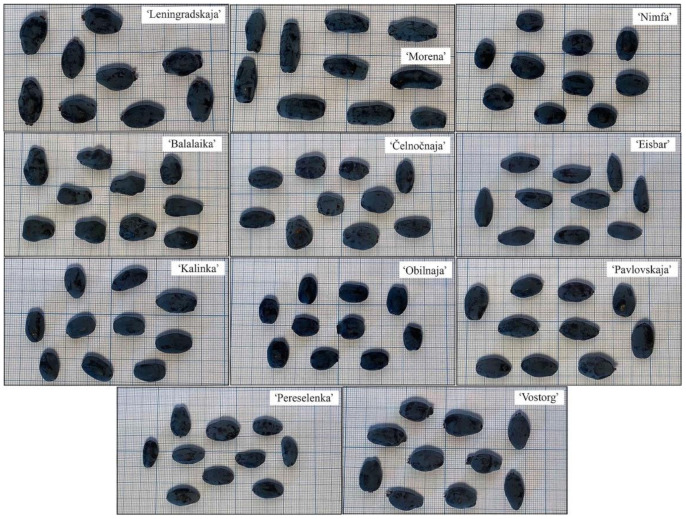
Berry shape and relative size of *Lonicera caerulea* cultivars at the stage of full maturity.

Statistically significant differences were determined between cultivars in terms of berry weight, length, and width (Figure 1, Table 1). The average berry weight was 0.76 ± 0.03 g, with ‘Morena’ (late ripening cv.) and ‘Pereselenka’ (medium-early cv.) distinguished by the heaviest berries: 1.05 ± 0.05 g and 0.96 ± 0.03 g, respectively (Table 1). No correlation was observed between berry size and group of cultivars by ripening time. According to a study carried out in Slovenia [20], the weight of 50 berries ranged from 45.13 to 90.59 g, which corresponds to 0.90–1.81 g per berry. However, they studied different cultivars in different climatic conditions. The collection of the genus *Lonicera* L. investigated in this study was located at an altitude 76 m above sea level, WGS84 coordinates 54.87054° N, 23.91620° E, while Senica et al. presented results obtained in Vučetinec location (46°26′22″ N; 16°22′15″ E) at an altitude 270 m above sea level [20]. Some weather for the location of our study, Kaunas, is as follows: hottest month, July (18 °C avg.); coldest month, January (−3 °C avg.); annual precipitation is 343.3 mm. For Vučetinec, these data are as follows: hottest month, July (22 °C avg.); coldest month, January (2 °C avg.); annual precipitation, 841.3 mm.

Certainly, berry size and weight are highly dependent not only on the intrinsic characteristics of cultivars, but on local meteorological conditions, yield size set and berry maturity stage, among other factors.

In freshly pressed berries, the pH was 3.40 on average (Table 2). Berries of ‘Leningradskaja’ showed the lowest acidity (pH 3.61), meanwhile the highest acidity was in ‘Morena’ (pH 3.235). In a study conducted in Switzerland by Auzanneau et al. [9], the pH of freshly pressed berries was 3.0 on average and varied slightly from year to year.

In this study, a considerably high DM content was observed in ‘Pavlovskaja’. Meanwhile, ‘Kalinka’, ‘Nimfa’, and ‘Morena’ exhibited significantly lower DM contents (Table 2). The average DM content of berries of all studied cultivars amounted to 14.21 ± 0.12%. These results are close to those obtained by Senica et al. [20], who reported the highest DM content of 16%. Meanwhile, Gerbrandt et al. [8] reported DM variation in blue honeysuckle berries within the range of 13.1 to 18.2%, because their research included not only Russian but Japanese and Kuril genotypes as well.

**Figure 2:**
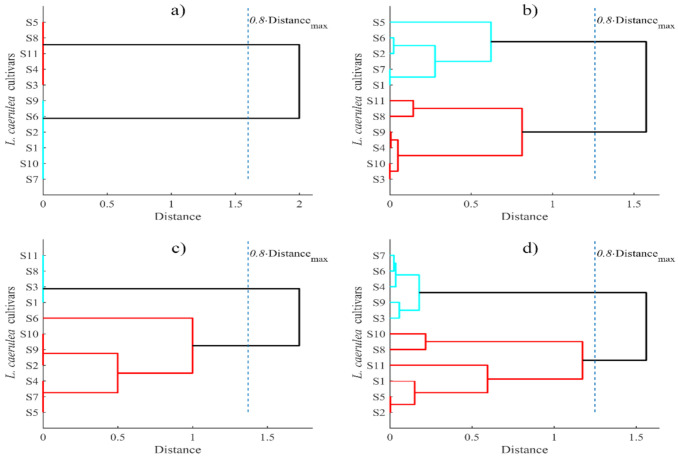
Grouping of *Lonicera caerulea* cultivars according to the biochemical composition: (**a**) pH and DM; (**b**) total phenolic compounds content, total anthocyanins content, and DPPH radical scavenging activity; (**c**) saccharides; (**d**) organic acids and vitamin C (sample codes are listed in Table 1).

The dendrogram, cut at 0.8 of the maximum observed distance, has revealed two groups of cultivars by pH and DM contents (Figure 2a).

### 2.2. Total Phenolic Content (TPC), Total Anthocyanin Content (TAC) and Radical Scavenging Capacity (RSC)

The TPC in berries of honeysuckle amounted to 590.3 ± 4.4 mg/100 g fresh weight (FW) on average. Considerable variation in TPC was observed between the studied cultivars. It ranged from 364.02 ± 0.41 mg/100 g in ‘Vostorg’ to 784.5 ± 0.3 mg/ 100 g in ‘Obilnaja’ (Table 3). Liu et al. [30] reported the average amount of TPC as 654.8 mg/100 g. Meanwhile, according to a study carried out in Canada, the total phenolic content in honeysuckle berries ranged from 634 to 1154 mg GAE/100 g fresh weight (FW), with the mean value of 832 mg GAE/100 g FW [28]. Thus, our study confirmed some lower TPC levels. Interestingly, Raudsepp et al. [23] highlighted the value of honeysuckle berries by stating that their TPC are higher than those of blackcurrant and bilberry.

The average TAC expressed in cyanidin-3-glucoside (the predominant anthocyanin) equivalent was 317.6 ± 5.0 mg/100 g (Table 3). A lower amount of these pigments expressed through cyanidin-3-glucoside (235.4 mg/100 g) was reported from Poland [5]. From the study in Canada, it was found that TAC varied between 70 and 314 mg/100 g FW expressed in cyanidin-3-O-glucoside equivalents [28]. Our study showed that anthocyanins comprised 53.8% of the total phenolic content on average. The literature data on qualitative composition of anthocyanins showed cyanidin 3-glucoside making up 84.77% of the total anthocyanin content in blue honeysuckle fruit [31]. This is in accordance with some other literature data, reporting that cyanidin-3-glucoside comprises up to 80–90% of the total anthocyanins in berries of blue honeysuckle [9]. In general, we assume that differences in TPC and TAC are largely caused by the genetic differences of the cultivars. Certain distinctive differences between cultivars were also reported in a comprehensive review of studies on blue honeysuckle by Gołba et al. [4].

Regarding the antioxidant effects, the highest DPPH radical scavenging capacity was characteristic for the cultivars ‘Vostorg’ and ‘Eisbar’. The average RSC of all studied cultivars amounted to 356.1 ± 3.3 mg TE/100 g with relatively low variation between them, i.e., from 344.1 ± 8.2 to 377.3 ± 8.5 mg TE/100 g (Table 3).

Hierarchical clustering analysis was carried out to establish similarity of the cultivars regarding TPC, TAC and radical scavenging capacity (Figure 2b). The dendrogram showed two clusters, and confirmed that there were no significant differences between ‘Obilnaja’ (S10) and ‘Balalaika’ (S3), ‘Pereselenka’ (S4) and ‘Kalinka’ (S9) in one cluster, as well as between ‘Eisbar’ (S1) and ‘Pavlovskaja’ (S7) in the other cluster in terms of total TPC, TAC, and antioxidant capacity.

### 2.3. Contents of Saccharides

Sugar content is an important property which determines the taste of berries and influences their attractiveness to consumers. Two monosaccharides, fructose and glucose, were predominant in berries of blue honeysuckle cultivars (Table 4). Our study showed significant differences in the contents of fructose and glucose between cultivars, varying from 2.44 ± 0.013 to 4.68 ± 0.096 and from 2.51 ± 0.013 to 4.41 ± 0.090 g/100 g, respectively.

The average amounts of fructose and glucose were 3.23 ± 0.02 and 3.16 ± 0.02 g/100 g, respectively. The highest contents of both fructose and glucose were obtained in ‘Leningradskaja’, while the lowest was in ‘Kalinka’. Sucrose was not detected in berries of five cultivars, while the other six cultivars accumulated relatively small amounts of this disaccharide. Rupasinghe et al. [21,28] also noted that blue honeysuckle berries accumulate only insignificant amounts of sucrose; monosaccharides accounted for 95% of the total sugars. Hierarchical cluster analysis categorized the cultivars studied into three groups (‘Eisbar’, ‘Balalaika’, ‘Nimfa’, and ‘Leningradskaja’; ‘Čelnočnaja’, ‘Kalinka’, and ‘Obilnaja’; ‘Vosotorg’, ‘Pavlovskaja’, and ‘Pereselenka’) in terms of saccharide contents, except for the cultivar ‘Morena’ (Figure 2c).

### 2.4. Contents of Organic Acids and Vitamin C

In this study, the accumulation of citric, malic, and quinic acids was analyzed. Significantly different amounts of the organic acids were established between cultivars (Table 5).

The citric acid was predominant and ranged from 543.03 ± 11.10 mg/100 g in ‘Leningradskaja’ to 979.87 ± 5.70 mg/100 g in ‘Morena’. On average, honeysuckle berries accumulated 769.41 ± 5.34 g/100 g citric acid. Meanwhile, some lower average amounts of malic and quinic acids were established, i.e., 289.90 ± 2.64 mg/100 g and 45.00 ± 0.37 mg/100 g, respectively.

Rupasinghe et al. [28] reported the dominance of citric acid, accounting for 30–58% of the total organic acid content in the berries of blue honeysuckle. In the other study, citric acid was also reported as the most abundant; however, malic acid and quinic acid levels were established as lower [20]. The reason of the differences might have been different genotypes studied and environmental factors. 

The importance of berries in human diets is also determined by the amount of vitamins they provide; vitamin C, or ascorbic acid, is particularly valued. According to our data, the content of ascorbic acid depended on cultivar properties (Table 5). Between cultivars, the contents of ascorbic acid varied from 14.55 ± 0.30 to 53.58 ± 0.45 mg/100 g with an average of 34.26 ± 0.25 mg/100 g.

As Auzanneau et al. reported, ascorbic acid was in the range of 1.78–4.21 mg/g DM [9]. Another study reported that ascorbic acid contents in berries of blue honeysuckle varied from 31.9 to 44.5 mg/100 g depending on cultivar and year [5], which coincides with the results obtained by our study. Meanwhile, Plekhanova and Streltsyna reported some bigger differences in the contents of ascorbic acid, falling into a range of 30.5–103.5 mg/100 g [32].

Cluster analysis subdivided the studied cultivars into two groups. In each group, two pairs of the most similar cultivars were placed in terms of organic acid and ascorbic acid contents (Figure 2d).

### 2.5. Antibacterial Activity of Aqueous and Ethanolic Extracts

In this study, stronger inhibition effects of ethanolic extracts were established against all test cultures (Table 6). All strains of bacteria tested were less susceptible to aqueous extracts of blue honeysuckle berries. Among the tested cultures, *Enterococcus faecalis* was the most susceptible to both ethanol and aqueous extracts, with average inhibition zones of 21.40 and 18.54 mm, respectively, whereas the lowest zones of inhibition were observed for *Escherichia coli* culture, with averages of 16.33 and 9.40 mm, respectively. Molina et al. [24] reported that ethanolic extracts of blue honeysuckle berries demonstrated growth inhibition of *Listeria monocytogenes*, *E. coli*, *Staphylococcus aureus*, and *L. monocytogenes* bacterial strains, which is in accordance with our results. Results of some other studies have also shown antimicrobial activity of the phenolic fraction on *Candida parapsilosis, Staphylococcus epidermidis*, *Escherichia coli*, *Enterococcus faecalis*, and *Streptococcus mutans* [17].

**Figure 3:**
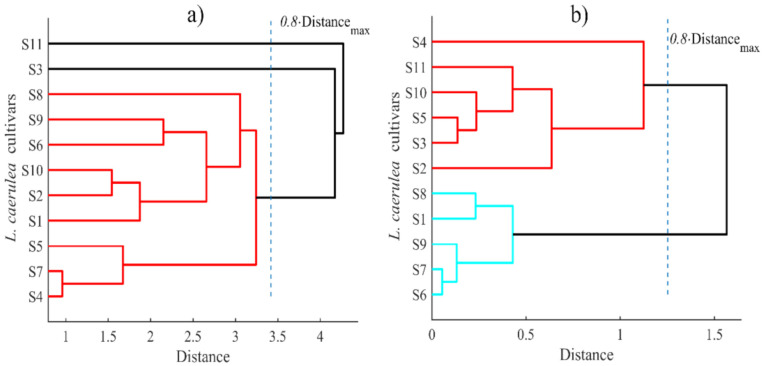
Grouping of *Lonicera caerulea* cultivars according to the antibacterial activity: (**a**) ethanolic extracts; (**b**) aqueous extracts (sample codes are listed in Table 1).

The dendrogram of hierarchical clustering analysis cut at 0.8 of the maximum observed distance revealed that with ethanolic extracts, nine cultivars showed similar patterns of antibacterial effects against the bacteria tested; only ‘Leningradskaja’ and ‘Balalaika’ fell apart (S11 and S3 samples, respectively, Figure 3a).

Correlation analysis revealed some relationships between the contents of bioactive compounds and inhibition of bacterial activity (Table 7).

The data on antibacterial activity of ethanolic extracts corroborated negative correlations between TPC and the growth of *B. subtilis*, *S. aureus*, and *P. aeruginosa*, and also between TAC and the growth of *B. subtilis*, *P. aeruginosa* and *S. enterica*. Comparable results were reported by some other studies, where negative correlations were also observed between TPC and TAC levels and activities of *S. aureus* and *P. aeruginosa* [33], or correlation was not significant [34]. We can presume that the stronger antibacterial effect of ethanol extracts was due to other biologically active compounds.

In aqueous extracts, the observed strong correlation between the contents of citric and quinic acids and Gram-positive bacterium *B. subtilis* inhibition zones (with correlation coefficients 0.768 and 0.705, respectively) revealed that these acids might have impacted on the growth of *B. subtilis*. Some weaker correlations were observed between malic acid and Gram-positive *S. aureus*, and Gram-negative *E. coli* and *P. aeruginosa* (correlation coefficients 0.373, 0.488 and 0.546, respectively): inhibition zone increased along with the increase of malic acid content.

Only *E. faecalis* was susceptible to the content of monosaccharides; particularly, glucose and fructose (see Table 7), while higher amounts of fructose demonstrated negative correlations with inhibition zones of *B. subtilis*, *S. aureus* and *P. aeruginosa*. In general, there were no clear differences on susceptibility patterns of Gram-positive and Gram-negative bacteria in this study. Interestingly, berries of cultivars with high contents of fructose accumulated higher contents of glucose (correlation coefficient 0.958). Contents of all saccharides studied decreased with increasing amounts of citric and quinic acids (Table 7). These results suggest that different bioactive compounds might be responsible for antibacterial activity in aqueous and ethanolic extracts of *L. caerulea* berries.

## 3. Materials and Methods

### 3.1. Plant Material

Eleven honeysuckle cultivars—‘Eisbar’, ‘Čelnočnaja’, ‘Balalaika’, ‘Pereselenka’, ‘Vostorg’, ‘Morena’, ‘Pavlovskaja’, ‘Nimfa’, ‘Kalinka’, ‘Obilnaja’, and ‘Leningradskaja’—were selected from the genetic resource collection of the Botanical Garden of Vytautas Magnus University. The collection is located in the central part of Lithuania, WGS84 coordinates 54.87054° N, 23.91620° E, at an altitude of 76 m above sea level. Duration of the growing season here is about 180 days. Berries were sampled from three randomly chosen shrubs in each cultivar in May–June at the stage of full ripening. The ripening stage was determined by visual evaluation of fruit for the blue color of berries and the brown color of seeds. The length and width dimensions of berries were obtained by using digital calipers, with an accuracy of 0.1 mm. Berry weight was determined by the analytical balance (model DJ-150E, ISHIDA Co., Ltd., Kyoto, Japan) with an accuracy of 0.01 g. For each cultivar, three replications of 50 berries were used. All samples were kept in plastic bags in a freezer at a temperature of −28 °C until analyses. The berries were removed from the freezer, and after 30 min were homogenized with a blender (Bosch MSM16500). Dry matter (DM) content was determined by drying 10 g of crushed berries at 102 °C to a constant weight (TCF 50, Argolab, Carpi, Italy). For pH measurements in crushed berries, a pH-meter (Denver Instrument Company, Bohemia, NY, USA) was used. Different extracts were prepared from homogenized berries for the analyses. For each cultivar, three replication samples were prepared.

### 3.2. Chemicals

Malic acid (Sigma-Aldrich, Darmstadt, Germany), citric acid (Sigma-Aldrich, Germany), quinic acid (Sigma-Aldrich, Germany), hydrochloric acid fixanal (Sigma-Aldrich, Germany), oxalic acid (Sigma-Aldrich, Germany), fructose (Sigma-Aldrich, Germany), glucose (Sigma-Aldrich, Germany) and sucrose (Sigma-Aldrich, Germany) HPLC-grade solvents were used for analysis. Acetonitrile (ACN) and trifluoroacetic acid (TFA) were obtained from Sigma-Aldrich (St. Louis, MO, USA). Ethanol (Riedel-de-Haën, Seelze, Germany) and vitamin C powder, 100% purity, were obtained from the Myprotein Co. (The Hut Group, Cheshire, UK). Folin–Ciocalteu reagent, gallic acid, 2,2-diphenyl-1-picrylhydrazyl radical (DPPH) and 6(±)-Hydroxy-2,5,7,8-tetramethylchromane-2-carboxylic acid (Trolox) were purchased from Sigma-Aldrich (Germany). Carrez I solution and Carrez II solution were prepared by dissolving 3.6 g of potassium hexacyanoferrate (II) in 100 mL of distilled water and 7.2 g of zinc sulphate in 100 mL of distilled water, respectively.

### 3.3. Preparation of Extracts

Aqueous extracts for antimicrobial activity were prepared by extracting 2 g of berry paste with 8 mL of water in an ultrasonic bath (Ultrasonix cleaner proclean 3.0DSP) for 30 min at frequency 37 kHz, temperature 28–37 °C. It was then centrifuged for 10 min at 5300 rpm (Labofuge 200 Heraeus Thermo Scientific, Waltham, MA, USA, Rotor 3760, 2700× *g*). The supernatant was used for the evaluation of antibacterial activity. 

Ethanolic extracts were prepared by extracting 2 g of berry paste with 15 mL of 95% (*v*/*v*) food grade ethanol acidified with 0.1 N HCl in an ultrasonic bath (Ultrasonix cleaner proclean 3.0DSP) for 20 min. The obtained extract was decanted, and a new 15 mL portion of solvent was used. Extraction was repeated three times, all three extracts were combined, and volume was adjusted to 50 mL with acidified ethanol (pH 1.2). This extract was used for total phenolics, total anthocyanins content, antibacterial activity, and radical scavenging capacity evaluation.

Aqueous extracts for the determination of saccharides were prepared as follows: 2 g of berry paste were extracted with 60 mL of water in a water bath at 60 °C for 30 min (GFL No-1092, Burgwedel, Germany), then clarified with Carrez I and Carrez II solution, diluted to 100 mL with water, and filtered through a paper filter followed by a 0.22 µm pore size membrane filter.

Aqueous extracts for the determination of organic acids were prepared by the extracting 5 g of berry paste were extracted with 20 mL of water in an ultrasonic bath for 20 min. Then, they were clarified with Carrez I and Carrez II solution, diluted to 25 mL with water, and filtered through a paper filter followed by a 0.22 µm pore size membrane filter.

Extracts for vitamin C determination were prepared according to slightly modified method of Auzanneaau et al. [9]. For this purpose, 5 g of berry paste were extracted with 10 mL of oxalic acid solution (10 g/L) in an ultrasonic bath for 20 min. After centrifugation for 10 min at 5300 rpm (2700× *g*), the obtained supernatant was filtered through a paper filter and then through a 0.22 µm pore size membrane filter.

### 3.4. Chemical Analysis

#### 3.4.1. Determination of Total Anthocyanin Content (TAC)

The absorption of ethanolic extract 1:10 diluted with acidified (0.1 N HCl, *v*/*v*) ethanol was measured on a spectrophotometer Genesys-5 (Thermo Spectronic, Rochester, USA) at 535 nm. The concentration of TAC was determined from the calibration curve, which was constructed by measuring the absorption of cyanidin-3-glucoside (MW 449.4, ε = 26.900) reference solution [35].

#### 3.4.2. Determination of Total Phenolic Content (TPC)

The TPC was measured with Folin–Ciocalteu reagent, as originally described by Singleton et al. [36]. Briefly, 30 μL of sample were mixed with 150 μL of 10-fold diluted (*v*/*v*) with distilled water and Folin−Ciocalteu reagent (1:10), and 120 μL of 7.5% Na_2_CO_3_. After the mixing of all reagents, the microplate was placed in the reader and shaken for 30 s. After incubation for 30 min at room temperature, the absorbance of the mixtures was measured at 765 nm. All measurements were performed in triplicate. A blank sample, which was prepared daily, contained the same amount of distilled water. A series of gallic acid solutions in the concentration range of 0.025–0.35 mg/mL was used for the calibration curve. The results were expressed in mg of gallic acid equivalents (GAE) per 100 g of fresh weight. The regression equation *y* = 9.8307*x* + 0.1215 (*R*^2^ = 0.9987) was used. 

#### 3.4.3. Determination of DPPH Radical Scavenging Capacity (RSC)

The RSC of extracts against stable DPPH^•^ was determined by a slightly modified spectrophotometric method using a 96-well microplate reader (FLUOstar Omega (BMG LABTECH, Ortenberg, Germany). DPPH working solution was prepared in methanol (0.0024 g/100 mL) and diluted with methanol to an absorbance of 0.7–0.8 at 515 nm. For each well, an aliquot of 7.5 μL extract was mixed with 300 μL of DPPH. The decrease in absorbance was measured at 515 nm by comparing with a blank sample. The final RSC values were calculated by a regression equation (*y* = 340.62*x* + 7.8965, *R*^2^ = 0.99) of Trolox concentration. The antioxidant capacity of each sample is expressed as mg of Trolox equivalent (TE) per 100 g of fresh weight. 

#### 3.4.4. Determination of Organic Acids

Separation by analytical RP-HPLC was performed using the Shimadzu Prominence analytical HPLC system with an SPD-M20A diode array detector set at 220 nm, an autosampler, and LC Solutions software (Shimadzu Corp., Kyoto, Japan). The Hydrosphere C18 (5 μm, 12 nm, 150 × 4.6 I.D., YMC Co., Ltd., Kyoto, Japan) column was used with a temperature of 30 °C. The eluent was 20 mM Na_2_HPO_4_ buffer solution, pH adjusted to 2.8 with acetic acid. The flow rate was 1.0 mL/min. The injection volume was 20 μL. Calibration curves of quinic, citric, and malic acid were used for the quantification; concentration range was from 1 to 20 mg/mL.

#### 3.4.5. Determination of Saccharides

Separation conditions were as follows: the eluent was a mixture of 75 parts by volume of acetonitrile and 25 parts by volume water, flow rate was 1.2 mL/min, and 20 μL was injected. The YMC-Pack Polyamine II 250 × 4.6 mm, 5 μm (YMC Co., Ltd., Japan) column was used with a temperature of 28 °C. Detection was performed using an Evaporative Light Scattering Detector ELSD-LTII (Shimadzu Corp., Japan). Calibration curves of fructose, glucose and sucrose were used for the quantification, concentration range was from 1 to 25 mg/mL.

#### 3.4.6. Determination of Vitamin C

A Shimadzu Prominence series (Shimadzu corp., Kyoto, Japan) HPLC system with Atlantis dC18 5 µm 4.6 × 150 mm column (Waters, Dublin, Ireland) was used for the separation and quantification of vitamin C. Mobile phase A—0.1% TFA in H_2_O; B—0.1% TFA in ACN. Time program: B conc. 0% → 3% (0.0–5.0 min) → 15% (6.0 min) → 20% (10.0 min) → 100% (12.0 min) → 100% (25.0 min). Flow rate, 1.4 mL/min. Column temperature 30 °C, injection volume 20 μL. Vitamin C was recorded at 210 nm using an SPD-M20A diode array detector (Shimadzu Corp., Kyoto Japan). Quantification of vitamin content was performed using a calibration curve of standard solutions, concentration range was from 0.1 to 1 mg/mL. 

### 3.5. Study of Antibacterial Activity

The antibacterial activity of ethanolic and aqueous berry extracts were examined by the agar well diffusion method according to the methodology described by Bobinaitė et al. [37]. Gram-positive bacteria *Bacillus subtilis* subsp. *spizizenii* ATCC 6633, *Staphylococcus aureus* subsp. *aureus* ATCC 25923, *Enterococcus faecalis* ATCC 19433 and Gram-negative bacteria *Pseudomonas aeruginosa* ATCC 27853, *Salmonella enterica* subsp. *enterica* serovar *Typhimurium* ATCC14028, and *Escherichia coli* ATCC 25922 were tested. These strains were grown in peptone–soy bouillon (LAB 04; LabM. Ltd., Heywood, UK) for 24 h at 37 °C. After cultivation, culture cells were mixed using a mini shaker MS 1 (Wilmington, NC, USA) and the cell suspensions were adjusted according to McFarland No 0.5 standard [38]. The suspension of bacteria cells was introduced into a dissolved plate count agar *Liofilchem* (LD 610040) medium cooled to 47 °C, and 10 mL of the suspension were pipetted into a 90 mm diameter Petri plate. Wells of diameter 8 mm were punched in the agar and filled with 50 µL of extracts. The plates were incubated overnight at 37 °C. The *B. subtilis* plates were incubated overnight at 30 °C. After incubation, the inhibition zones were measured with the digital calipers to an accuracy of 0.01 mm, and the average zone was calculated as a mean of three replications. Berry paste has been used to prepare ethanolic extracts and ethanol was diluted with juice, whereas as a control in the blank sample for ethanolic and aqueous extracts, ethanol (80%) and distilled water were used.

### 3.6. Statistical Analysis

The input data matrix for statistical analysis comprised 24 measured quantities of 11 tested blue honeysuckle cultivars: antibacterial properties of ethanolic and aqueous extracts against six bacterial strains, total phenolic compound composition (TPC), radical scavenging activity (DPPH), anthocyanins, pH, dry matter content, ascorbic acid, citric acid, malic acid, quinic acid, fructose, glucose, and sucrose. Statistical analysis was performed using MATLAB software (The MathWorks, Inc., Natick, MA, USA, version R2016b (9.1.0), 64-bit). The data analysis included correlation analysis, one-way ANOVA, and hierarchical cluster analysis (HCA). Only statistically significant (*p* ≤ 0.05) correlation coefficients are presented in the correlation matrix. The same level of significance was used in hypotheses testing for differences between means by employing one-way ANOVA with multiple (pairwise) comparison procedure applying Tukey’s HSD test. 

Clustering of honeysuckle cultivars by their characteristics was performed using hierarchical cluster analysis. The HCA as an unsupervised clustering algorithm using Euclidean distance was employed to create clusters according to the underlying input data structure estimated using specific similarity measure. The obtained dendrograms were cut at a chosen level (i.e., 0.8 of a maximum observed distance) to form groups of cultivars with similar characteristics. The input data matrix was divided into 6 submatrices, which were used in HCA dendrogram construction in order to analyze similarity of the cultivars regarding any of the specified set of properties: (i) antibacterial properties of aqueous extracts; (ii) antibacterial properties of ethanolic extracts; (iii) total content of phenolic compounds, radical scavenging activity and anthocyanins; (iv) pH and dry matter content; (v) ascorbic, citric, malic and quinic acids; and (vi) fructose, glucose and sucrose.

## 4. Conclusions

The profiles of major bioactive compounds, DPPH antioxidant capacity, and antimicrobial activity of ethanolic and aqueous extracts of berries were established for 11 blue honeysuckle (*L. caerulea*) cultivars. ‘Obilnaja’ was distinguished by the highest content of total phenolics exceeding the average of all cultivars by 33%, while ‘Nimfa’ showed the highest content of total anthocyanins, exceeding the average by 24%. Taken more broadly, ‘Leningradskaja’ should be acknowledged as the cultivar possessing not only a high total anthocyanin content but also accumulating the highest levels of fructose and glucose which, combined with the low total content of organic acids, provides a sweet berry taste. The study also revealed that some cultivars, namely, ‘Kalinka’, ‘Morena’, and ‘Čelnočnaja’, are among the best berry sources of organic acids and vitamin C. Investigation of antimicrobial impact of ethanolic extracts showed that the total amount of phenolic compounds and total anthocyanins did not reduce the growth of both Gram-positive and Gram-negative bacteria tested. Examination of the aqueous extracts corroborated the finding that malic acid was the most potential inhibitor of Gram-positive *S. aureus* and Gram-negative *E. coli* and *P. aeruginosa.* All cultivars showed high antioxidant activities evaluated by DPPH assays with the lowest variation of this property between cultivars if compared to the other studied properties.

In general, the results of the study suggest that berries of the studied 11 blue honeysuckle cultivars present themselves as rich potential food sources with health-promoting properties, and that differences in the contents of bioactive compounds as well as antioxidant and antibacterial activities are largely predefined by the genotype.

## Figures and Tables

**Table 1:** Ripening time, berry weight and size characteristics (means ± SD) of *Lonicera caerulea* cultivars.

Cultivar Name	Sample No.	Ripening Time	* Berry Weight, g	* Berry Length, cm	* Berry Width, cm
‘Eisbar’	S1	medium-early	0.79 ± 0.03 ^d^	2.38 ± 0.20 ^d^	0.92 ± 0.08 ^c^
‘Čelnočnaja’	S2	medium-early	0.89 ± 0.02 ^d^	1.88 ± 0.09 ^c^	0.97 ± 0.03 ^c^
‘Balalaika’	S3	early	0.75 ± 0.03 ^c^	1.71 ± 0.12 ^bc^	1.07 ± 0.11 ^c^
‘Pereselenka’	S4	medium-early	0.96 ± 0.03 ^e^	1.90 ± 0.10 ^d^	1.08 ± 0.05 ^c^
‘Vostorg’	S5	late	0.70 ± 0.02 ^c^	1.72 ± 0.07 ^bc^	0.97 ± 0.08 ^bc^
‘Morena’	S6	late	1.05 ± 0.05 ^f^	2.63 ± 0.47 ^d^	0.94 ± 0.11 ^bc^
‘Pavlovskaja’	S7	medium-early	0.87 ± 0.01 ^d^	1.85 ± 0.06 ^c^	1.03 ± 0.03 ^c^
‘Nimfa’	S8	late	0.58 ± 0.02 ^b^	1.45 ± 0.05 ^b^	0.87 ± 0.06 ^b^
‘Kalinka’	S9	late	0.54 ± 0.01 ^b^	1.41 ± 0.17 ^b^	0.88 ± 0.03 ^b^
‘Obilnaja’	S10	medium-early	0.46 ± 0.01 ^a^	1.31 ± 0.11 ^a^	0.83 ± 0.08 ^a^
‘Leningradskaja’	S11	medium-early	0.75 ± 0.01 ^c^	1.81 ± 0.08 ^bc^	1.07 ± 0.04 ^c^
Average			0.76 ± 0.03	1.82 ± 0.02	0.97 ± 0.02

* Different letters denote statistically significant differences between means within columns (ANOVA using Tukey’s HSD, *p* ≤ 0.05).

**Table 2:** Berry pH values and dry matter contents (means ± SD) of *Lonicera caerulea* cultivars.

Cultivar Name	* pH	* Dry Matter, %
‘Eisbar’	3.33 ± 0.01 ^b^	13.98 ± 0.12 ^d^
‘Čelnočnaja’	3.43 ± 0.01 ^d^	15.67 ± 0.10 ^g^
‘Balalaika’	3.47 ± 0.035 ^e^	13.84 ± 0.12 ^d^
‘Pereselenka’	3.42 ± 0.01 ^d^	14.25 ± 0.10 ^e^
‘Vostorg’	3.39 ± 0.01 ^c^	13.31 ± 0.02 ^c^
‘Morena’	3.24 ± 0.01 ^a^	13.17 ± 0.08 ^bc^
‘Pavlovskaja’	3.54 ± 0.014 ^f^	16.57 ± 0.19 ^h^
‘Nimfa’	3.43 ± 0.02 ^d^	13.11 ± 0.03 ^b^
‘Kalinka’	3.24 ± 0.01 ^a^	12.89 ± 0.07 ^a^
‘Obilnaja’	3.42 ± 0.01 ^cd^	14.33 ± 0.01 ^e^
‘Leningradskaja’	3.61 ± 0.01 ^g^	15.28 ± 0.31 ^f^
Average	3.40 ± 0.03	14.21 ± 0.12

* Different letters denote statistically significant differences between means within columns (ANOVA using Tukey’s HSD, *p* ≤ 0.05).

**Table 3:** Total phenolic content (TPC), total anthocyanin content (TAC), their ratio (TAC/TPC), and DPPH^•^ radical scavenging capacity (RSC) (means ± SD) in berries of *Lonicera caerulea* cultivars.

Cultivar Name	* TPC	* TAC	TAC/TPC	* RSC
	mg GAE/100 g FW	mg/100 g FW	%	mg TE/100 g FW
‘Eisbar’	603.2 ± 5.0 ^f^	304.3 ± 1.1 ^b^	50.4	371.8 ± 5.4 ^c^
‘Čelnočnaja’	515.9 ± 3.2 ^d^	277.8 ± 1.1 ^a^	53.8	350.6 ± 8.4 ^ab^
‘Balalaika’	647.8 ± 5.7 ^h^	324.4 ± 7.2 ^c^	50.1	352.9 ± 8.6 ^ab^
‘Pereselenka’	463.6 ± 3.4 ^b^	283.0 ± 7.7 ^a^	61.1	344.4 ± 8.5 ^a^
‘Vostorg’	364.0 ± 0.4 ^a^	316.1 ± 5.0 ^bc^	86.8	377.3 ± 8.5 ^c^
‘Morena’	694.8 ± 4.0 ^i^	308.4 ± 6.1 ^b^	44.4	363.9 ± 3.1 ^bc^
‘Pavlovskaja’	494.1 ± 5.8 ^c^	284.1 ± 6.6 ^a^	57.5	353.8 ± 6.2 ^ab^
‘Nimfa’	624.8 ± 1.5 ^g^	394.1 ± 8.4 ^e^	63.1	361.7 ± 1.1 ^bc^
‘Kalinka’	750.5 ± 3.9 ^j^	316.2 ± 4.8 ^bc^	42.1	347.9 ± 7.6 ^ab^
‘Obilnaja’	784.5 ± 0.3 ^k^	343.9 ± 3.7 ^d^	43.8	348.6 ± 3.3 ^ab^
‘Leningradskaja’	549.9 ± 11.2 ^e^	341.0 ± 4.1 ^d^	62.0	344.1 ± 8.2 ^a^
Average	590.3 ± 4.4	317.6 ± 5.0	53.8	356.1 ± 3.3

* Different letters denote statistically significant differences between means within columns (ANOVA using Tukey’s HSD, *p* ≤ 0.05).

**Table 4:** Contents of saccharides (means ± SD) in berries of *Lonicera caerulea* cultivars.

Cultivar Name	* Fructose	* Glucose	** Sucrose
	g/100 g FW	g/100 g FW	g/100 g FW
‘Eisbar’	3.13 ± 0.03 ^e^	3.26 ± 0.03 ^g^	0
Čelnočnaja’	3.57 ± 0.02 ^i^	3.57 ± 0.02 ^h^	1.24 ± 0.01 ^d^
‘Balalaika’	3.20 ± 0.03 ^f^	3.17 ± 0.03 ^ef^	0
‘Pereselenka’	3.49 ± 0.03 ^h^	3.22 ± 0.02 ^fg^	1.35 ± 0.01 ^f^
‘Vostorg’	3.43 ± 0.00 ^g^	3.14 ± 0.00 ^e^	1.02 ± 0.00 ^b^
‘Morena’	2.77 ± 0.02 ^c^	2.62 ± 0.02 ^b^	0
‘Pavlovskaja’	3.13 ± 0.04 ^e^	2.93 ± 0.03 ^c^	1.32 ± 0.02 ^e^
‘Nimfa’	2.67 ± 0.01 ^b^	2.93 ± 0.01 ^c^	0
‘Kalinka’	2.44 ± 0.01 ^a^	2.51 ± 0.01 ^a^	0
‘Obilnaja’	3.03 ± 0.00 ^d^	2.99 ± 0.00 ^d^	1.04 ± 0.00 ^c^
‘Leningradskaja’	4.68 ± 0.10 ^j^	4.41 ± 0.10 ^i^	1.03 ± 0.02 ^c^
Average	3.23 ± 0.02	3.16 ± 0.02	1.17 ± 0.01 **

* Different letters denote statistically significant differences between means within columns (ANOVA using Tukey’s HSD, *p* ≤ 0.05). ** Average of 6 cultivars.

**Table 5:** Contents of organic acids and vitamin C (means ± SD) in berries of *Lonicera caerulea* cultivars.

Cultivar Name	* Citric Acid	* Malic Acid	* Quinic Acid	* Vitamin C
	mg/100 g FW	mg/100 g FW	mg/100 g FW	mg/100 g FW
‘Eisbar’	688.39 ± 5.75 ^d^	338.88 ± 2.83 ^f^	28.76 ± 0.24 ^b^	53.58 ± 0.45 ^k^
‘Čelnočnaja’	574.20 ± 3.51 ^b^	389.48±2.38 ^h^	23.31 ± 0.14 ^a^	40.94 ± 0.25 ^h^
‘Balalaika’	729.43 ± 6.42 ^f^	135.75±1.19 ^a^	44.82 ± 0.39 ^e^	18.85 ± 10.17 ^c^
‘Pereselenka’	711.51 ± 5.17 ^e^	175.21±1.27 ^b^	44.51 ± 0.32 ^e^	35.12 ± 0.26 ^f^
‘Vostorg’	607.02 ± 0.69 ^c^	313.10 ± 0.35 ^d^	28.25 ± 0.03 ^b^	34.07 ± 0.04 ^e^
‘Morena’	979.87 ± 6.05 ^j^	281.82 ± 1.64 ^c^	74.02 ± 0.43 ^i^	53.09 ± 0.31 ^j^
‘Pavlovskaja’	763.53 ± 8.97 ^g^	171.32 ± 2.01 ^b^	60.66 ± 0.71 ^g^	40.41 ± 0.47 ^g^
‘Nimfa’	977.55 ± 2.30 ^j^	335.02 ± 0.79 ^e^	53.30 ± 0.13 ^f^	17.23 ± 0.04 ^b^
‘Kalinka’	952.47 ± 5.68 ^i^	343.13 ± 1.78 ^f^	71.67 ± 0.37 ^h^	47.99 ± 0.25 i
‘Obilnaja’	936.48 ± 0.34 ^h^	364.28 ± 0.13 ^g^	33.46 ± 0.01 ^d^	21.03 ± 0.01 ^d^
‘Leningradskaja’	543.03 ± 11.10 ^a^	340.78 ± 6.97 ^f^	32.28 ± 0.66 ^c^	14.55 ± 0.30 ^a^
Average	769.41 ± 5.34	289.90 ± 2.64	45.00 ± 0.37	34.26 ± 0.25

* Different letters denote statistically significant differences between means within columns (ANOVA using Tukey’s HSD, *p* ≤ 0.05).

**Table 6:** Antibacterial activity of ethanolic and aqueous berry extracts of *L. caerulea* cultivars, presented as mean inhibition zone diameter ± SD in mm (*n* = 3) including well diameter (8 mm).

Cultivar Name	Extract Type	Bacteria					
		* *Bacillus subtilis*	* *Staphylococcus aureus*	* *Escherichia coli*	* *Pseudomonas aeruginosa*	* *Salmonella enterica*	* *Enterococcus faecalis*
‘Eisbar’	ethanolic	17.00 ± 0.00 ^cde^	18.00 ± 0.81 ^efg^	17.00 ± 0.00 ^bcd^	18.33 ± 0.47 ^e^	16.66 ± 0.47 ^bc^	20.00 ± 0.00 ^a^
	aqueous	12.00 ± 0.00 ^c^	9.00 ± 0.00 ^b^	9.00 ± 0.00 ^b^	10.33 ± 0.47 ^bc^	9.00 ± 0.00 ^b^	18.00 ± 0.00 ^c^
‘Čelnočnaja’	ethanolic	16.66 ± 0.47 ^bcd^	17.33 ± 0.47 ^de^	15.33 ± 1.24 ^b^	17.33 ± 0.00 ^cd^	17.00 ± 0.00 ^def^	20.66 ± 0.47 ^ab^
	aqueous	11.00 ± 0.00 ^b^	12.00 ± 0.81 ^e^	10.00 ± 0.00 ^e^	11.33 ± 0.47 ^f^	9.00 ± 0.00 ^d^	19.00 ± 0.00 ^e^
‘Balalaika’	ethanolic	15.00 ± 0.00 ^a^	17.50 ± 0.81 ^def^	13.00 ± 0.00 ^a^	15.33 ± 0.47 ^a^	16.66 ± 0.47 ^cde^	20.00 ± 0.00 ^a^
	aqueous	11.00 ± 0.00 ^b^	11.33 ± 0.81 ^de^	9.00 ± 0.00 ^b^	10.33 ± 0.47 ^bc^	9.00 ± 0.00 ^b^	20.00 ± 0.00 ^e^
‘Pereselenka’	ethanolic	17.66 ± 0.47 ^de^	19.33 ± 0.47 ^g^	16.33 ± 1.24 ^bcd^	18.00 ± 0.00 ^de^	17.00 ± 0.00 ^def^	22.66 ± 0.47 ^cd^
	aqueous	11.00 ± 0.00 ^b^	0	0	0	9.00 ± 0.00 ^b^	19.00 ± 0.00 ^d^
‘Vostorg’	ethanolic	18.00 ± 0.00 ^e^	18.00 ± 0.81 ^efg^	18.00 ± 0.00 ^cd^	19.33 ± 0.47 ^f^	16.66 ± 0.47 ^cde^	22.00 ± 0.00 ^c^
	aqueous	10.00 ± 0.00 ^a^	9.00 ± 0.00 ^b^	9.00 ± 0.00 ^b^	9.33 ± 0.47 ^b^	9.00 ± 0.00 ^b^	20.00 ± 0.00 ^e^
‘Morena’	ethanolic	15.66 ± 0.47 ^ab^	15.33 ± 0.47 ^bc^	17.33 ± 1.24 ^d^	17.00 ± 0.00 ^bc^	18.00 ± 0.00 ^f^	20.66 ± 0.47 ^ab^
	aqueous	13.00 ± 0.00 ^d^	14.33 ± 0.81 ^f^	11.00 ± 0.00 ^d^	13.33 ± 0.47 ^e^	10.66 ± 0.47 ^c^	16.00 ± 0.00 ^a^
‘Pavlovskaja’	ethanolic	17.00 ± 0.00 ^cde^	19.00 ± 0.81 ^fg^	17.00 ± 0.00 ^bcd^	18.33 ± 0.47 ^b^	17.66 ± 0.47 ^ef^	23.00 ± 0.00 ^d^
	aqueous	12.00 ± 0.00 ^c^	10.00 ± 0.00 ^bc^	10.00 ± 0.00 ^c^	10.33 ± 0.47 ^bc^	9.00 ± 0.00 ^b^	17.00 ± 0.00 ^b^
‘Nimfa’	ethanolic	15.66 ± 0.47 ^ab^	18.33 ± 0.47 ^efg^	16.33 ± 1.24 ^b^	17.00 ± 0.00 ^bc^	14.00 ± 0.00 ^a^	20.66 ± 0.47 ^ab^
	aqueous	13.00 ± 0.00 ^d^	11.00 ± 0.00 ^cd^	9.00 ± 0.00 ^b^	12.33 ± 0.47 ^de^	9.66 ± 0.47 ^b^	19.00 ± 0.00 ^d^
‘Kalinka’	ethanolic	16.00 ± 0.00 ^abc^	14.00 ± 0.81 ^ab^	16.00 ± 0.00 ^bc^	16.33 ± 0.47 ^b^	16.66 ± 0.47 ^cde^	21.00 ± 0.00 ^b^
	aqueous	12.00 ± 0.00 ^c^	12.00 ± 0.00 ^de^	9.00 ± 0.00 ^b^	11.33 ± 0.47 ^cd^	10.66 ± 0.47 ^c^	18.00 ± 0.00 ^c^
‘Obilnaja’	ethanolic	17.66 ± 0.47 ^de^	16.33 ± 0.47 ^cd^	17.33 ± 1.24 ^bcd^	17.00 ± 0.00 ^bc^	16.00 ± 0.00 ^cd^	20.66 ± 0.47 ^ab^
	aqueous	11.00 ± 0.00 ^b^	11.00 ± 0.00 ^cd^	9.00 ± 0.00 ^b^	11.33 ± 0.47 ^cd^	9,00 ± 0.00 ^b^	19.00 ± 0.00 ^d^
‘Leningradskaja’	ethanolic	15.00 ± 0.00 ^a^	13.00 ± 0.81 ^a^	16.00 ± 0.00 ^bc^	17.33 ± 0.47 ^cd^	14.66 ± 0.47 ^ab^	24.00 ± 0.00 ^e^
	aqueous	10.00 ± 0.00 ^a^	9.33 ± 0.47 ^b^	9.00 ± 0.00 ^b^	9.33 ± 0.47 ^b^	0	19.00 ± 0.00 ^d^

* Different letters denote statistically significant differences between means within columns (ANOVA using Tukey’s HSD, *p* ≤ 0.05). When an inhibition zone was not observed, the result was estimated as 0.

**Table 7:** Correlation matrix between tested berry variables (TPC, total phenolic content; TAC, total anthocyanins content; RSC, radical scavenging capacity) * of 11 *Lonicera caerulea* cultivars.

		TPC	TAC	RSC	pH	Dry Matter	Vitamin C	Citric Acid	Malic Acid	Quinic Acid	Fructose	Glucose	Sucrose
**TPC**		1.000											
**TAC**		0.385	1.000										
**RSC**				1.000									
**pH**		−0.425		−0.329	1.000								
**Dry Matter**		−0.362	−0.448	−0.365	0.723	1.000							
**Vitamin C**			−0.644	0.309	−0.692		1.000						
**Citric Acid**		0.735	0.422		−0.564	−0.525		1.000					
**Malic Acid**									1.000				
**Quinic Acid**		0.422			−0.438	−0.309	0.317	0.738	−0.325	1.000			
**Fructose**		−0.513			0.728	0.540	−0.413	−0.833		−0.622	1.000		
**Glucose**		−0.403			0.726	0.506	−0.467	−0.802		−0.690	0.958	1.000	
**Sucrose**		−0.584	−0.427	−0.306	0.586	0.717		−0.538		−0.445	0.579	0.440	1.000
**Inhibition of bacteria by ethanolic extracts**	***B. subtilis***	−0.356	−0.356							-			
	***S. aureus***	−0.503											
	***E. coli***												
	***P. aeruginosa***	−0.697	−0.298	0.363						-			
	***S. enterica***		−0.734										
	***E. faecalis***	−0.498											
**Inhibition of bacteria by aqueous extracts**	***B. subtilis***							0.768		0.705	−0.761	−0.672	−0.611
	***S. aureus***							0.335	0.373		−0.324		−0.474
	***E. coli***								0.488				
	***P. aeruginosa***								0.546				
	***S. enterica***		-								−0.551	−0.507	
	***E. faecalis***							−0.536		−0.737	0.393	0.469	

* Only statistically significant (*p* ≤ 0.05) coefficients of correlation are presented.
